# Alemtuzumab for refractory primary systemic vasculitis—a randomised controlled dose ranging clinical trial of efficacy and safety (ALEVIATE)

**DOI:** 10.1186/s13075-022-02761-6

**Published:** 2022-04-01

**Authors:** Seerapani Gopaluni, Rona Smith, Donna Goymer, Hugh Cahill, Elizabeth Broadhurst, Elizabeth Wallin, Mark McClure, Afzal Chaudhry, David Jayne

**Affiliations:** 1 University of Cambridge, Box 118, Addenbrooke’s Hospital, Hills Road, Cambridge, CB20QQ UK; 2 Cambridge University Hospitals NHS Foundation Trust, Cambridge, UK

**Keywords:** ANCA, Alemtuzumab, Vasculitis, Behçet’s disease, Clinical trial, T cells, Lymphocytes

## Abstract

**Background:**

Primary systemic vasculitis (PSV) is a heterogeneous group of autoimmune conditions. There is an unmet need for alternative therapies that lead to sustained remission in patients with refractory disease. Alemtuzumab, an anti-CD52 antibody, depletes lymphocytes for prolonged periods and, in retrospective studies, has induced sustained, treatment-free remissions in patients with refractory/relapsing vasculitis but has raised safety concerns of infection and secondary autoimmunity. This phase IIb clinical trial aimed to assess the efficacy and safety of alemtuzumab, at two different doses, in inducing remission in refractory vasculitis patients.

**Methods:**

The ALEVIATE trial was a randomised, prospective, open-label, dose ranging clinical trial. Patients with refractory ANCA-associated vasculitis (AAV) or Behçet’s disease (BD) were randomised to receive either 60 mg or 30 mg alemtuzumab. Treatments were administered at baseline and 6 months or earlier where clinically appropriate. A maximum of three treatments were allowed within the 12-month study period.

**Results:**

Twenty-three patients received at least one dose of alemtuzumab. Twelve had AAV, and 11 a diagnosis of BD. The median age was 40 years (range 28–44), with a prior disease duration of 61 months (42–103). Sixteen (70%) achieved either complete (6/23, 26%) or partial (10/23, 44%) response at 6 months. Eight (35%) maintained remission to the end of the trial without relapse. Ten severe adverse events were observed in 7 (30%) patients; 4 were related to alemtuzumab. There were no differences in clinical endpoints between the 60 and 30 mg alemtuzumab treatment groups.

**Conclusion:**

In a selected group of refractory vasculitis patients, alemtuzumab led to remission in two thirds of patients at 6 months. Remission was maintained to 12 months in a third of the patients, and the safety profile was acceptable.

**Trial registration:**

ClinicalTrials.gov identifier: NCT01405807, EudraCT Number: 2009-017087-17. Registered on April 07, 2011.

**Supplementary Information:**

The online version contains supplementary material available at 10.1186/s13075-022-02761-6.

## Background

Primary systemic vasculitis (PSV) encompasses a group of autoimmune conditions, characterised by inflammation, neutrophilic infiltration, endothelial cell swelling and fibrinoid necrosis of blood vessels. PSV can affect multiple systems and lead to organ dysfunction.

Anti-neutrophil cytoplasmic antibody (ANCA)-associated vasculitis (AAV), a form of small vessel vasculitis, comprises granulomatosis with polyangiitis (GPA), microscopic polyangiitis (MPA) and eosinophilic granulomatosis with polyangiitis (EGPA) and is one of the most common forms of PSV, but the spectrum is broad. Behçet’s disease (BD) is a type of variable vessel vasculitis and was included in the Nomenclature of Vasculitides at the 2012 Chapel Hill Consensus Conference [1].

Untreated, AAV has a 93% mortality within 2 years primarily due to pulmonary and renal failure [2]. The introduction of immunosuppressive treatment in the form of glucocorticoids, cyclophosphamide and more recently biologic agents has transformed survival [3]. Despite induction of remission in the majority, a proportion of patients (10–20%) inadequately respond to this therapy, and relapsing disease is common, with over 50% of patients experiencing a relapse within 5 years [4–6]. Relapse is associated with increased exposure to immunosuppressive medications and glucocorticoids with their associated risks and progressive accrual of disease-related damage [7].

BD is a heterogeneous condition, and in severe cases, organ or life-threatening vascular, neurological or gastrointestinal manifestations can occur. Tumour necrosis factor (TNF) blockade has transformed management of individuals with severe BD [8], although there remains a subset of patients with ongoing refractory disease [9]. Currently, there is no consensus in the management of anti-TNF failures in BD, and alemtuzumab is suggested in UK treatment pathways for refractory cases (http://www.behcets.nhs.uk/download/behcets-drug-pathway/).

Autoreactive T cells contribute to the immune dysregulation and inflammation that causes organ damage in PSV [10, 11]. Alemtuzumab, a humanised monoclonal anti-CD52 antibody, reversibly depletes lymphocytes, monocytes and eosinophils, but there are prolonged changes in the reconstituted B cell and T cell repertoires, with a sustained reduction in CD4+ T cells except for regulatory T cells which increase [12]. It is an approved therapy for lymphoma and has been used off label in transplantation.

A study of 71 patients with refractory or relapsing AAV treated compassionately with alemtuzumab demonstrated remission in 60 (85%), 24 of whom (40%) remained in remission for over 1 year and 10 (17%) for at least 3 years [13]. This study identified age more than 50 years, dialysis dependency and severe infection at the time of treatment as risk factors for early mortality. In a retrospective study of BD patients treated with alemtuzumab, 26/32 patients (81%) achieved complete or partial remission [14, 15]. Similar to the AAV study, relapses were common in BD patients (47% at 1 year). In these historic BD cohorts, three different dosing regimens were used, totalling 134 mg, 95 mg or 60 mg. Subsequent relapse was associated with lower total alemtuzumab dosing: relapse rates were 22% in the 134 mg group versus 62% and 63% respectively in the lower dose groups observed at 12 months.

Alemtuzumab is a licensed therapy for multiple sclerosis, where the labelled dose is 60 mg (12 mg/day on 5 consecutive days, 60 mg total dose) repeated at 12 months (12 mg/day on 3 consecutive days, 36 mg total dose), but there is a paucity of data in other autoimmune indications or of the effects of different dosing regimens [16, 17].

There is an unmet need for alternative therapies, with an acceptable safety profile, that lead to remission in patients with relapsing or refractory PSV. Prior experience has identified AAV and BD as forms of PSV likely to benefit from alemtuzumab. Even though the clinical phenotype and therapeutic strategies of these diseases differ, T cell dysregulation is at the core of the pathogenesis for both diseases. Alemtuzumab with its pan-lymphocyte activity may help to restore the immune homeostasis. Conducting studies in rare diseases is challenging, and with the advances in therapeutic strategies, the number of patients with refractory diseases is small. However, the effects of refractory diseases are devastating for the patients. Some compromises in trial methodology are necessary to overcome the challenges of recruiting refractory vasculitis patients.

The ALEVIATE trial was designed to test the hypotheses that alemtuzumab induces remission in vasculitis patients that have failed to respond to conventional therapies and that alemtuzumab has an acceptable safety profile in selected refractory or relapsing AAV and BD patients. Furthermore, the trial explored the effect of two dosing regimens on clinical and biomarker end-points.

## Methods

### Trial design and patient eligibility

The ALEVIATE trial was a randomised, single-centre, open-label, phase IIb, dose ranging clinical trial of alemtuzumab in refractory PSV, conducted at the Cambridge University Hospitals NHS Foundation Trust between June 2010 and May 2018. Patients were recruited from a specialised vasculitis clinic, and all provided written informed consent.

Key inclusion criteria were a diagnosis of GPA, MPA, EGPA or BD and active vasculitis with at least one severe or three non-severe items as recorded in the Birmingham Vasculitis Activity Score for Wegener’s granulomatosis (BVAS/WG). A GPA or MPA diagnosis required typical clinical features and confirmatory histology or proteinase 3 (PR3) or myeloperoxidase (MPO)-ANCA positivity; the EGPA patients met the 1990 American College of Rheumatology (ACR) classification criteria, whilst those with BD met either the ISG (International Study Group) or ICBD (International Criteria for Behçet’s Disease) diagnostic criteria. For AAV patients, previous induction therapy with either cyclophosphamide or methotrexate or rituximab, in combination with prednisolone for at least 3 months, and for BD at least 6 months of anti-tumour necrosis factor (TNF) therapy was required for inclusion. Background immunosuppression with immunomodulators such as azathioprine and mycophenolate mofetil was stopped before recruitment to the trial. Refractory disease reflected disease activity despite induction therapy or relapse whilst receiving relapse prevention therapy.

Exclusion criteria were age < 18 years or > 60 years; serum creatinine > 150 μmol/L; total white cell count < 4 × 10^9^/L or lymphocyte count < 0.5 × 10^9^/L or neutrophil count < 1.5 × 10^9^/L; lung haemorrhage with hypoxia; previous alemtuzumab therapy at any time; rituximab in the last 6 months or anti-TNF therapy in the last month; active infection with HIV, hepatitis B or C; or other systemic infections.

### Treatments

Patients were randomised to receive either low dose (LD) 30 mg (15 mg/day for two consecutive days) or high dose (HD) 60 mg (30 mg/day for two consecutive days) of alemtuzumab by intravenous (IV) infusion. The treatment allocation was determined using the minimisation procedure of Pocock and Simon. The minimisation was balanced by ANCA type (MPO, PR3 and negative). The course was repeated at 6 months, but the interval could be reduced to 3 months for uncontrolled or relapsing disease. Patients were allowed to have a maximum of 3 courses of alemtuzumab over the trial period of 1 year for incomplete response or relapse with a minimum gap of 3 months between treatments and only if the total lymphocyte count had recovered to more than 0.3 × 10^9^/L.

All patients received 500 mg of IV methylprednisolone, 10 mg chlorpheniramine and 1 g paracetamol before each infusion, for infusion reaction prophylaxis. All patients received sulphamethoxazole/trimethoprim 480 mg daily and aciclovir 200 mg twice a day as *pneumocystis jiruvecii* pneumonia (*PJP*) and herpes prophylaxis respectively, for at least 3 months following alemtuzumab or until their CD4 count was more than 0.2 × 10^9^/L. Nystatin 1 ml four times daily was prescribed for 2 months following each infusion. Oral prednisolone at a maximum dose of 10 mg/day was prescribed for the first month after entry with a standardised taper of 2.5 mg per month aiming to withdraw prednisolone by 4 months. Earlier withdrawal was permitted according to clinical disease activity, but those intolerant of steroid withdrawal due to adrenal suppression could remain on the minimum dose that controlled their symptoms. An increase in prednisolone to 30 mg/day for 1 week then reducing by 5 mg/week to protocol was permitted for persistent vasculitis disease or minor flares.

### Follow-up and assessments

Patients were assessed at 6-week intervals and followed for 1 year from enrolment into the trial or until withdrawal. Disease activity was assessed by BVAS/WG and, in addition, for BD patients, the Behçet’s disease current activity form (BDCAF) to document BD disease activity [18]. The combined damage assessment (CDA) was used to document ‘all cause’ damage accrual from the time of diagnosis, whilst the Short-Form 36 (SF-36) questionnaire was collected at baseline, 6 and 12 months to assess patient reported outcomes. At each visit full blood count, renal function tests, electrolytes, liver function tests, thyroid function tests and lymphocyte subsets were measured.

### Outcomes

The primary outcomes were:

1. The proportion of patients with a vasculitis response (complete or partial) at 6 months. Complete response (CR) was defined as a BVAS/WG* of zero and partial response (PR) as BVAS/WG score ≤ 50% of baseline with no severe items and
2. The proportion of patients with a serious adverse event (SAE).

* BVAS/WG was used as primary outcome measure for both disease groups for consistency. BVAS/WG is not validated for use with BD; however, all the items that were present in BDCAF and not represented in BVAS/WG were recorded in the ‘other’ items section of the BVAS/WG form when present.

Secondary outcomes were:

1. The proportion of patients with treatment failure (failure to achieve a vasculitis response (either complete or partial) by 6 months or a vasculitis relapse between 6 and 12 months)
2. Relapse (defined as the appearance or re-appearance of severe disease (major BVAS/WG item) or appearance or re-appearance of at least two minor BVAS/WG items)
3. Time to first relapse
4. Accrual of damage (according to the Combined Damage Assessment, CDA)
5. Non-severe adverse events
6. Glucocorticoid exposure.

### Immunophenotyping

Peripheral blood mononuclear cells (PBMC) were extracted from whole blood from all patients at time points 0, 1.5, 3, 4.5, 6, 7.5, 9 and 12 months. T cell and B cell panels were used to investigate T cell and B cell subsets and the changes associated within these subsets in relation to treatment. Briefly, PBMC were separated over a Ficoll gradient and were incubated with a live-dead stain initially followed by an FcR blocker. One million cells were aliquoted to 6 different polystyrene FACS tubes and incubated with fluorochrome master mixes for 20 min at 4^o^C. After the washing steps, single cell suspensions were acquired on BD Fortessa flow cytometer and analysed in FlowJo™ 10 software for Mac (TreeStar). Statistical analysis of flow cytometric data at entry visit was compared to 4.5 months visit data.

### Statistical analysis

The sample size was determined based on feasibility for this phase IIb study. Given the rarity of these conditions, it would not be practical to recruit a large number of patients to satisfy the power calculation requirements to discriminate the effect of different dosing regimens. Continuous variables were expressed as medians and interquartile ranges. Categorical variables were presented as percentages and frequencies. The chi-square test was used for categorical variables, and Student’s *t*-test or non-parametric Mann-Whitney test was used for continuous variables to compare differences between the groups. 95% confidence intervals were shown for proportions. Data was analysed using R software (R foundation for Statistical Computing, Vienna, Austria) version 3.1.2. A two-tailed *p* value of ≤ 0.05 was considered significant.

## Results

### Baseline demographics

Twenty-four patients were recruited (Supplementary Figure). Of these, 23 received at least one course of alemtuzumab. One was withdrawn prior to receiving alemtuzumab, after hyposplenism was identified and concerns about increased infection risk. This patient was not included in subsequent analyses. Of the 23 patients, 10 received HD, and 13 received LD therapy. Six (26%) patients, three from each group, were withdrawn due to progressive disease; there were no withdrawals due to adverse events. One patient was lost to follow-up after 9 months in trial.

The median age was 41 years (range 28–44), with a prior disease duration of 61 months (range 42–103) (Table 1 and Table 2). Twelve had a diagnosis of AAV (8 GPA, 1 MPA, 3 EGPA and 11 of BD).

**Table 1:** Baseline demographics

	All subjects (*n* = 23)	Low dose (*n* = 13)	High dose (*n* = 10)
Age in years (median; IQR)	40.5 (28.0–44.0)	35.0 (25.0–46.5)	41.0 (33.0–44.0)
Sex (M to F)	8:15	6:7	2:8
Disease (AAV to BD)	12:11	6:7	6:4
GPA	8	4	4
MPA	1	1	0
EGPA	3	1	2
BD	11	7	4
BVAS/WG score at entry (median; IQR)	4.0 (4.0–5.5)	4.0 (4.0–6.0)	4.5 (4.0–5.0)
Prior disease duration in months (median; IQR)	61.0 (42.0–102.5)	76.0 (52.0–115.0)	51.0 (40.0–68.5)
Number of previous treatments received (median; IQR)	4 (3–5)	5 (3–5)	4(3–4.75)
ANCA specificity (PR3:MPO:Neg)	7:1:15	4:1:8	3:0:7
Prior median cumulative cyclophosphamide dose (AAV only) in grams (*n* = 12)	9.1 (7–19.4)	6.4 (4.2–25.8)	9.6 (8.7–17.3)
Prior median cumulative rituximab dose (AAV only) in grams (*n* = 12)	4.5 (3–5.75)	5 (3–6)	4 (3–5)

*AAV* ANCA-associated vasculitis, *BD* Behçet’s disease, *GPA* granulomatosis with polyangiitis, *MPA* microscopic polyangiitis, *eGPA* eosinophilic granulomatosis with polyangiitis, *IQR* interquartile range, *BVAS/WG* Birmingham Vasculitis Activity Score for Wegener’s granulomatosis, *PR3* proteinase 3, *MPO* myeloperoxidase

**Table 2:** Baseline demographics by disease sub-group and alemtuzumab dose received

	AAV (high dose) *N* = 6	AAV (low dose) *N* = 6		BD (high dose) *N* = 4	BD (low dose) *N* = 7
Age	41 (41–46)	42 (22–49)		39 (28–41)	29 (25–44)
Median disease duration in months	52 (38–95)	108 (52–109)		61 (50–71)	68 (16–108)
BVAS/WG score at entry (median; IQR)	4.5 (4.0–5.0)	5.0 (4.0–6.0)	BDCAF at entry (median, IQR)	10.5 (9.75–11.2)	8 (8–8)
BVAS/WG score 6 M	1 (0–1)	1 (0–3)	BDCAF score at 6 M	8 (6.5–9.25)	5 (0–8)
BVAS/WG score at 12 M	0 (0–2)	0 (0–0)	BDCAF score at 12 M	0 (0–3.5)	4.5 (0–9)

**Fig. 1:**
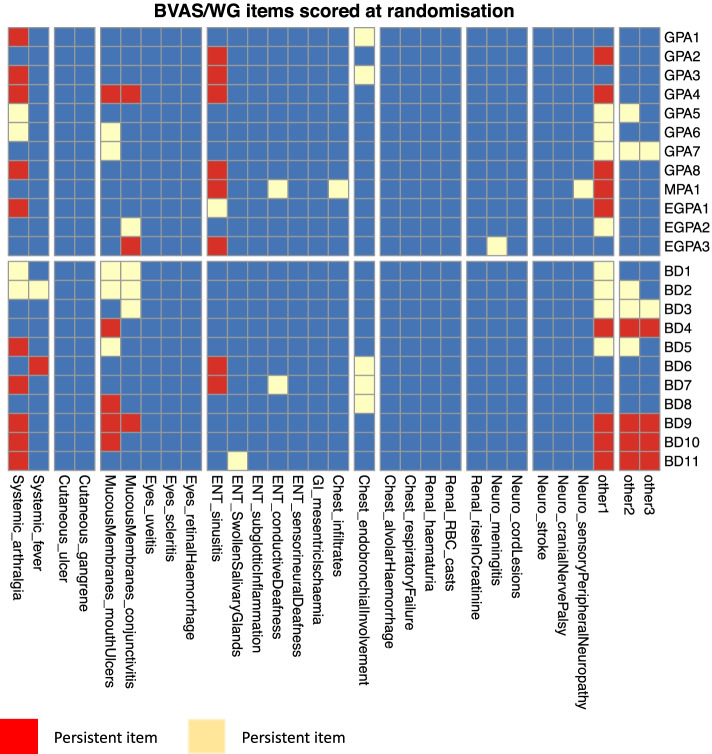
BVAS/WG items scored at randomisation

**Fig. 2:**
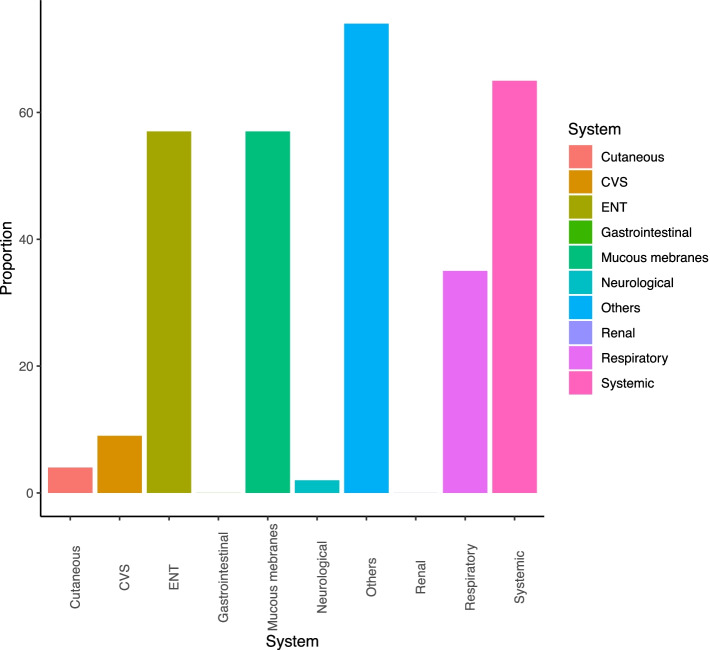
Proportion of patients with different organ system involvement at the time of recruitment into the trial

The involvement of different organ systems identified as new/worse or persistent on BVAS/WG scoring tool at the time of recruitment are shown in Figs. 1 and 2. Most of the patients had persistent disease that was not well controlled despite conventional therapy. None of the BD patients had manifestations of severe disease such as aneurysms or uveitis, whilst the majority of AAV patients had three or more minor items at the time of enrolment.

The median BVAS/WG score at entry was 4 (range 4–6), and the median number of prior immunosuppressive drugs received was 4 (range 3–5). All AAV patients had previously received cyclophosphamide or rituximab. Ten of 12 AAV patients (one with EGPA and one with MPA) had received cyclophosphamide (cumulative median dose of 9 g (range 7–19 g) and 11/12 rituximab (cumulative median dose of 5 g (range 3–6 g) previously. All BD patients had failed after anti-TNF agents and 10/11 after at least two anti-TNF agents sequentially used at least for 6 months (typically adalimumab subcutaneously 40 mg weekly or fortnightly or infliximab intravenous at 5 mg/kg every 4 weeks for 4 doses and then every 6 weeks or etanercept SC at 50 mg twice a week) with or without other immunomodulatory agents.

### Efficacy

**Fig. 3:**
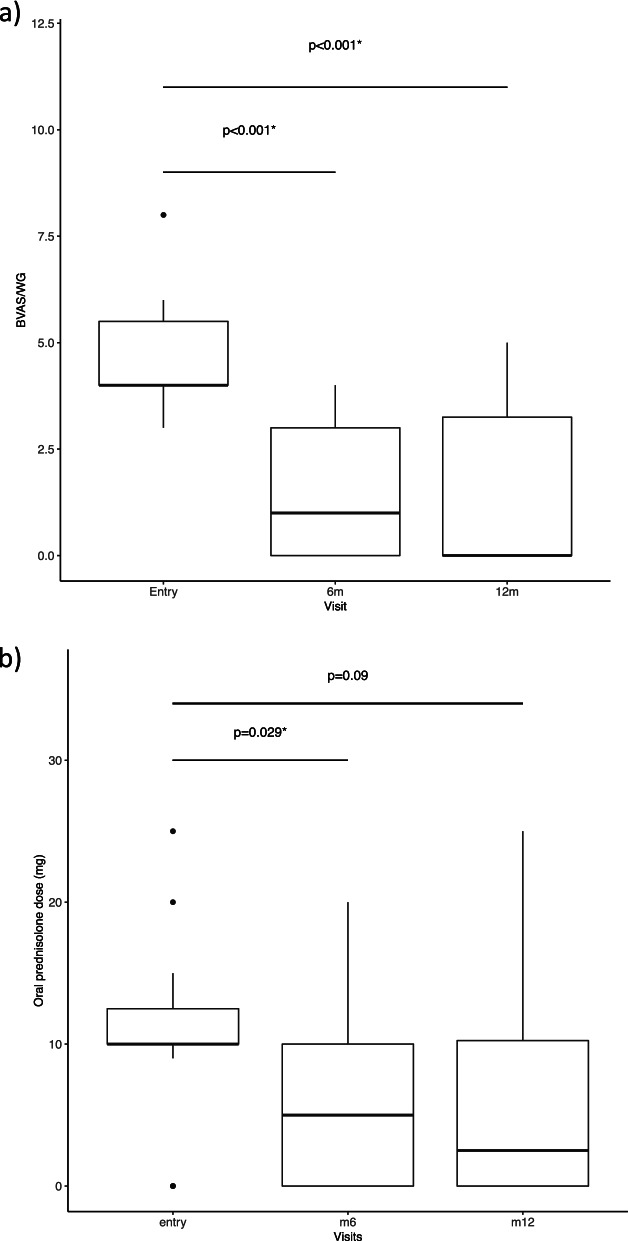
**a** Boxplot showing median and IQR of BVAS/WG scores at entry, 6 months and 12 moths. **b** Boxplot showing of oral prednisolone dose (in mg) at baseline, 6 months and 12 months

Sixteen patients (69.5%) achieved either complete (6/23, 26.1%) or partial (10/23, 43.5%) remission at 6 months (Table 3). Eight (34.7%) maintained remission to the end of the trial without relapse (Fig. 3). Seven of 10 (70.0%) in the HD and 9/13 (69.2%) in the LD groups were in remission at 6 months. The primary response definition did not take into account glucocorticoid dose. Eleven of 23 (47.8%) patients (5 in HD and 6 in LD groups) were in complete or partial remission at 6 months with a glucocorticoid dose of less than 7.5 mg/day.

**Table 3:** Response to alemtuzumab at 6 and 12 months

	AAV (***n*** = 12)	BD (***n*** = 11)	Total (***n*** = 23)
**At 6 months in CR**	4/12 (33.3%)	2/11 (18.2%)	6/23 (26.1%)
**At 6 months in PR**	5/12 (41.6%)	5/11 (45.5%)	10/23 (43.5%)
**At 6 months CR or PR**	9/12 (75%)	7/11 (63.6%)	16/23 (69.5%)
**At 12 months in CR**	5/12 (41.6%)	4/11 (36.4%)	9/23 (39.1%)
**At 12 months in PR**	0/12 (0%)	1/11 (9.1%)	1/23 (4.4%)
**At 12 months CR or PR**	5/12 (41.6%)	5/11 (45.5%)	10/23 (43.5%)

*CR* complete response, *PR* partial response, *AAV* ANCA-associated vasculitis, *BD* Behçet’s disease

Treatment failure, defined as failure to achieve a vasculitis response (complete or partial remission) by 6 months or vasculitis relapse between 6 and 12 months, occurred in 13/23 patients (56.5%). Of the 13, 6 were withdrawn (3 from each dose group; 2 with GPA, 2 with EGPA, 1 with MPA and 1 with BD) from the trial at 6 months due to progressive disease, and the other 7 (3 HD, 4 LD) (5 BD, 2 AAV) had a relapse between 6 and 12 months.

**Fig. 4:**
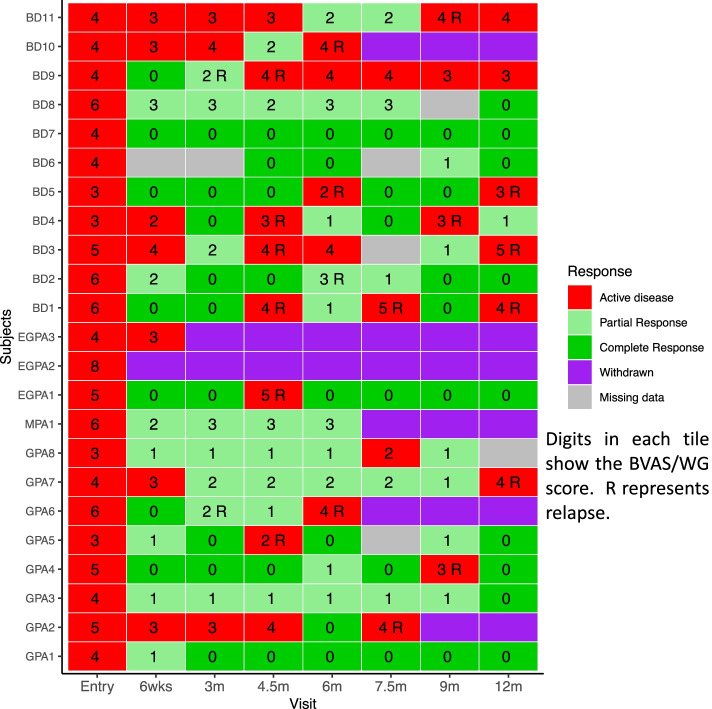
Individual response to therapy for each patient at each visit during the trial

Fourteen of 23 (60.9%) had at least one relapse during the 1-year follow-up period; the median time to relapse was 150 days (range 130–150). There was no difference in the risk of relapse (70.0% in HD and 53.8% of the patients in the LD groups) between the two dosing groups (log rank test, *p* = 0.48). Figure 4 depicts the individual disease course of each subject during the 1-year trial.

### Safety

There were 6 SAEs (low probability of being related to alemtuzumab) and 4 serious adverse reactions (SARs) (high probability of being related to alemtuzumab) in 7 patients (3 HD, 4 LD groups). Two SAEs occurred in one subject after enrolment but before the administration of alemtuzumab (myocarditis and skin infection—both events due to uncontrolled severe disease). There were 3 disease relapses and 1 admission due to palpitations (unrelated to alemtuzumab). The 4 SARs were as follows: infusion-related reaction (cytokine storm), viral gastroenteritis, cytomegalovirus (CMV) colitis and *Clostridium difficile* infection. The latter two events were of moderate to severe intensity and lasted for 19 and 25 days respectively. The patient with CMV colitis received two courses of alemtuzumab before the SAE and did not receive further courses. The patient with *Clostridium difficile* was withdrawn at a later time point due to progressive disease and did not receive further alemtuzumab. The patient with viral gastroenteritis had already received two courses of alemtuzumab, and the fourth patient with cytokine storm went on to receive a further course without any complications.

Non-severe adverse events were common (Table 4). Self-limiting short-lived infusion-related reactions were almost universal. In total, 93 non-serious adverse events were documented. Of these, 38 (40.8%) were minor infections, of which 63% were related to the respiratory system.

**Table 4:** Number and proportion of patients affected by adverse events

	All subjects (***n*** = 23)	Low dose (***N*** = 13)	High dose (***N*** = 10)
**Number (proportion) of patients with serious adverse events**	7 (30.4%)	4/13 (30.7%)	3/10 (30%)
**Total number of SAE/SAR**	10	8	2
**Number (proportion) of patients with non-serious adverse events**	23 (100%)	13 (100%)	10 (100%)
**Total number of non-serious adverse events**	93	54	39
**Total number (proportion) of non-serious infections**	38 (40.8%)	21 (38.8%)	17 (43.5%)

*SAE* severe adverse event, *SAR* severe adverse reaction

### Compliance with protocol

On average, each patient received a median of 2 (2–2.5) courses of alemtuzumab therapy during the 1-year trial. Patients in the HD group received a median cumulative dose of 120 mg (120–180 mg) and in the LD group 60 mg (60–60 mg) of alemtuzumab. Compliance with the planned glucocorticoid withdrawal by 6 months was poor. Only 7/23 (30.5%) patients had withdrawn glucocorticoids by 6 months. The median prednisolone dose on entry was 10 mg (range 10–12.5 mg), at 6 months was reduced to 5 mg (range 0–10 mg) (*p* = 0.029) and at 12 months to 2.5 mg (range 0–12.5 mg) (p = 0.09) (Fig. 3). The cumulative dose of steroids, including methylprednisolone, received during the 12 month trial was 4.2 g (± 1.7 g) for all subjects, 4.6(± 1.7 g) for HD group and 3.7(± 1.6 g) for the LD group (*P* = NS). The median daily dose of oral steroids (excluding 2000 mg of methyl prednisolone pre-infusion) given over the course of 1 year was 6 mg/day.

There were no changes in CDA scores or SF-36 questionnaire scores at 6 or 12 months compared to baseline or between the two dosing regimens. In terms of laboratory parameters, all patients had a reduction in total white cell count and lymphocyte count as expected following alemtuzumab treatment. Although there were trends to lower levels of CRP and ESR, most individuals started with low baseline levels. Thyroid stimulating hormone levels were normal in all but one patient throughout the trial, who went onto develop autoimmune thyroid disease during the 12 months follow-up period.

### T cell and B cell subset dynamics

**Fig. 5:**
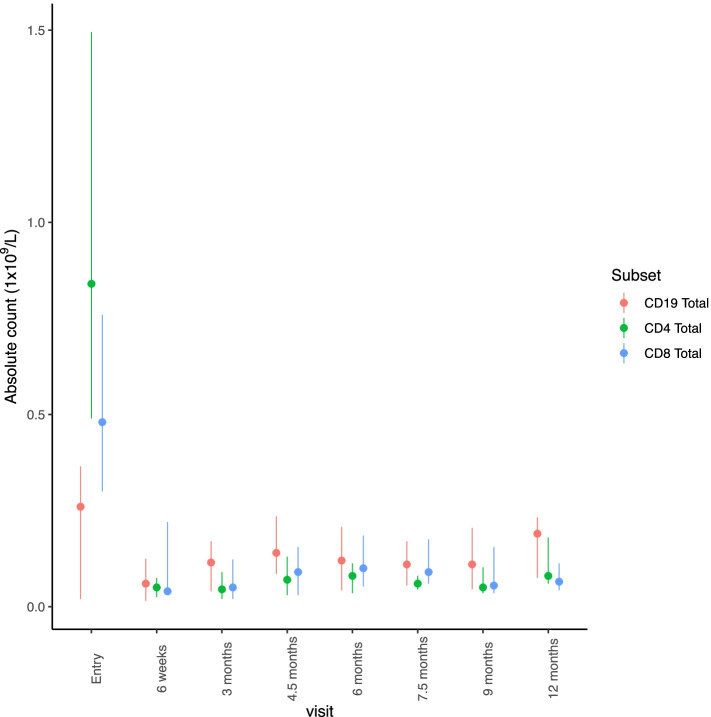
Absolute counts (1 × 10^9^/L) of CD4, CD8 and CD19 subsets tracked over the course of the trial. Plots represent median, lower and upper quartiles

Lymphocyte subsets were measured at all visits (Fig. 5). All patients receiving alemtuzumab depleted the lymphocytes, and there was no difference between the two dosing regimens in relation to lymphocyte counts at various time point. A single dose of alemtuzumab, irrespective of the dose, lead to significant and sustained reduction in both CD4 and CD8 subsets. There was a rise in the proportion of T regulatory cells (CD4 + CD127^lo^CD25^hi^) at 4.5 months when compared to the entry time point (median proportion at entry = 7.5% vs 14.85% at 4.5 M, *p* = 0.0007). In contrast to T cell subsets, B cell suppression was not sustained, and the recovery was quick. A majority of the returning B cells were of the naïve phenotype (65.2% vs 89.1% at entry and 4.5 months visit, *p* = 0.032). The dosing regimen had no effect on the level and duration of CD4, CD8 T cell and B cell suppression.

## Discussion

The potential for alemtuzumab to induce sustained remission in patients with refractory PSV, including AAV and BD was explored in a dose ranging study. Improvements in disease activity were seen in 70%, but only one third achieved sustained remission. No differences were observed between the two alemtuzumab doses. Alemtuzumab-related adverse events were common, but serious adverse event rates were no higher than seen with conventional therapies in this population [9, 19, 20].

The majority of individuals with BD and AAV will respond to standard immunosuppressive regimens including agents such as glucocorticoids, cyclophosphamide, rituximab and anti-TNF therapy [8, 9]. However, there remains an unmet need for the subset of individuals who pursue a frequently relapsing or refractory course. Alemtuzumab, a humanised anti-CD52 monoclonal antibody, manipulates both innate and adaptive immune systems, transiently depleting monocytes and eosinophils, as well as causing long-term alterations in the number and proportion of T and B cell subsets [12]. It is a licensed therapy for relapsing remitting multiple sclerosis and has also been used in small studies or for compassionate use in multiple autoimmune conditions [16, 17]. Retrospective cohort studies from our group have demonstrated efficacy in patients with AAV and BD, but adverse event rates were high, and there was a suggestion that reduced doses of alemtuzumab were associated with an increased risk of relapse [13–15]. The ALEVIATE trial was designed to assess the safety and efficacy of two doses of alemtuzumab as a second-line treatment for relapsing and refractory AAV and BD with patient selection adjusted to minimise risk.

The response rates for AAV patients in the ALEVIATE trial were similar to those reported from our previous compassionate use experience in refractory AAV disease and broadly equivalent to those seen with gusperimus and IVIg, although differences in trial design make comparison difficult [13–15, 20, 21]. Early relapses were common, although most responded to repeat alemtuzumab administration. No differences in efficacy or relapse rates were seen between high and low dose regimens in this small cohort, but this may have reflected the small sample size, and it is not known whether higher doses, above 60 mg, may have had a more durable effect.

Serious adverse events related to alemtuzumab occurred in 30% of patients; all resolved without long-term sequelae. Non-severe infections occurred commonly. The frequency and types of infection were similar to that seen in previous vasculitis studies with conventional agents [3, 22]. Prophylactic sulphamethoxazole/trimethoprim, aciclovir and nystatin were routinely administered, and there were no cases of herpes zoster or *PJP* pneumonia. Infusion reactions were common, despite premedication with intravenous methylprednisolone and chlorpheniramine. New onset autoimmunity, particularly thyroid disease, has been reported following alemtuzumab therapy. There was one case of asymptomatic hypothyroidism in this cohort, although 1 year of follow-up is likely to be too short to identify these typical later complications. Thus, the patient selection criteria in ALEVIATE, in particular, avoiding those aged over 60 years and those with a serum creatinine above 150umol/L, were validated, and concerns over safety should not be a barrier to alemtuzumab use in these selected populations.

This study has several limitations. Firstly, only a small number of patients were recruited into a single centre trial, and despite the refractory nature of disease, there was heterogeneity between individuals, both in terms of prior disease course and therapies received. None of the patients had severe organ threatening disease at the time of enrolment, and this restrictive inclusion criteria limit generalisability of the results. Furthermore, combining the data from two heterogenous disease cohorts would limit the generalisability of these results. However, as discussed previously, certain compromises in trial design are inevitable in the context of rare disease research.

Adherence to the glucocorticoid tapering regimen was poor, and only 30% of patients withdrew prednisolone, reflecting the refractory nature of disease in this population. However, there was reduction in the median prednisolone dose from 10 mg at baseline to 2.5 mg at 12 months. On average, patients received 4.2 g of prednisolone, and this may be adequate to partially treat non-severe manifestations of disease. This aspect may limit the interpretation of these results. The daily median dose of prednisolone was 6 mg/day (excluding intravenous methylprednisolone). As most of the patients had been on large doses of steroids for prolonged periods, it was difficult to completely withdraw steroids completely. However, it should be noted that this dose is half of what is typically given to treat AAV patients following induction and that these patients have previously failed to respond to high dose steroids.

The follow-up period was short, as the primary aim of the study was to demonstrate early efficacy and safety; we plan to collect long-term safety and efficacy follow-up data for these patients in order to understand the longer-term outcomes. Disease response was assessed by BVAS/WG which has not been validated in BD and in an open-label study may be subject to investigator bias. The study was designed prior to the extensive use of rituximab in AAV and the sequential use of anti-TNF therapy in BD, and as a result, the pool of patients with refractory vasculitis who were potentially eligible to enter this trial was smaller than anticipated. However, there is still a sub-group of patients who remain refractory to common treatment strategies and relapse frequently.

In the AAV subgroup, we observed a rapid rise in ANCA titres, following an initial fall in the majority of patients. This may be due to rapid early rebound in the B cell population following alemtuzumab therapy, partly driven by BAFF surge [23]. Rituximab was licensed in AAV after this trial was launched and has now become an effective, routine therapy for relapsing/refractory patients. Our results with alemtuzumab for the AAV subgroup are inferior to those with rituximab and do not support a role for alemtuzumab as an alternative to rituximab in current treatment guidelines [24–26]. Alemtuzumab has been used in the context of rituximab failure in AAV, but this should be evaluated on an individual patient basis with careful evaluation of infective risk. In contrast, there is no obvious therapy for anti-TNF failures in BD, with rituximab, cyclophosphamide and alemtuzumab being suggested for consideration in UK treatment pathways for refractory disease (http://www.behcets.nhs.uk/download/behcets-drug-pathway/). Although numbers are small, and the ALEVIATE study was not designed to separately evaluate disease subgroups, the results in the BD subgroup suggest that alemtuzumab could be a useful option for these patients and warrants further investigation.

The proposed mechanism of action of alemtuzumab is through depletion of autoreactive T and B cells, followed by an alteration in the repertoire of reconstituting cells, favouring a less autoreactive phenotype [12]. Given the increasing evidence of the role of the innate immune system in the pathogenesis of vasculitis, part of the early clinical efficacy of alemtuzumab may also be due to rapid depletion of innate immune cells such as monocytes [27].

Alemtuzumab administration led to severe depletion of the lymphocytes for prolonged periods irrespective of the dosing regimen. Within the T cell compartment, both CD4 and CD8 subsets were depleted for the entire duration of the trial. The proportion of T regulatory cells within the CD4 subset was higher after treatment when compared to baseline. This enrichment in T regulatory cells following alemtuzumab is in keeping with established literature [28]. However, B cell depletion was not sustained, and there is a rapid return towards baseline values. This rapid increase in B cells, majority of naïve phenotype, was also shown in multiple sclerosis patients treated with alemtuzumab. This was shown to be related to a surge in B cell activating factor (BAFF) following alemtuzumab administration [23]. Even though there was complete and prolonged depletion of lymphocytes peripherally, similar levels of depletion may not happen at the tissue level. This may partly explain variable and lack of sustained response seen in this cohort of patients.

We did not find a difference either in remission or relapse rates or adverse events between the two dosing regimens, but this study is underpowered to address this specific question. In addition, we did not identify any difference in the degree or duration of lymphocyte depletion between treatment groups.

## Conclusion

There is a need for alternative therapies to treat patients with refractory vasculitis that do not respond to conventional therapies. In the ALEVIATE trial, we explored the therapeutic benefits and safety aspects of alemtuzumab in patients with refractory vasculitis. In a selected group of refractory vasculitis patients, alemtuzumab led to remission in two thirds of patients at 6 months. Remission was maintained to 12 months in a third of the patients, and the safety profile was acceptable. However, the effect was not sustained, and relapses were common, necessitating repeat dosing and precluding the withdrawal of glucocorticoids in the majority of individuals.

## Supplementary Information

**Additional file 1: Supplementary Figure**. Consort diagram illustrating patient disposition throughout the trial.

## Data Availability

The datasets used and/or analysed during the current study are available from the corresponding author on reasonable request.

## Abbreviations

AAV: ANCA-associated vasculitis
ACR: American College of Rheumatology
ANCA: Anti-neutrophil cytoplasmic antibody
BD: Behçet’s disease
BDCAF: Behçet’s disease current activity form
BVAS/WG: Birmingham Vasculitis Activity Score for Wegener’s granulomatosis
CDA: Combined damage assessment
CR: Complete response
EGPA: Eosinophilic granulomatosis with polyangiitis
GPA: Granulomatosis with polyangiitis
HD: High dose
ICBD: International Criteria for Behçet’s Disease
ISG: International Study Group
IV: Intravenous
LD: Low dose
MPA: Microscopic polyangiitis
MPO: Myeloperoxidase
PBMC: Peripheral blood mononuclear cells
PR3: Proteinase 3
PR3: Partial response
PSV: Primary systemic vasculitis
SAE: Serious adverse event
SAR: Serious adverse reactions
SF36: Short-Form 36
TNF: Tumour necrosis factor
