# Supplementary figures and images for: Alemtuzumab for refractory primary systemic vasculitis—a randomised controlled dose ranging clinical trial of efficacy and safety (ALEVIATE)
Source: Arthritis Res Ther. 2022 Apr 1;24:81. doi: 10.1186/s13075-022-02761-6 (PMC8972754; doi:10.1186/s13075-022-02761-6)

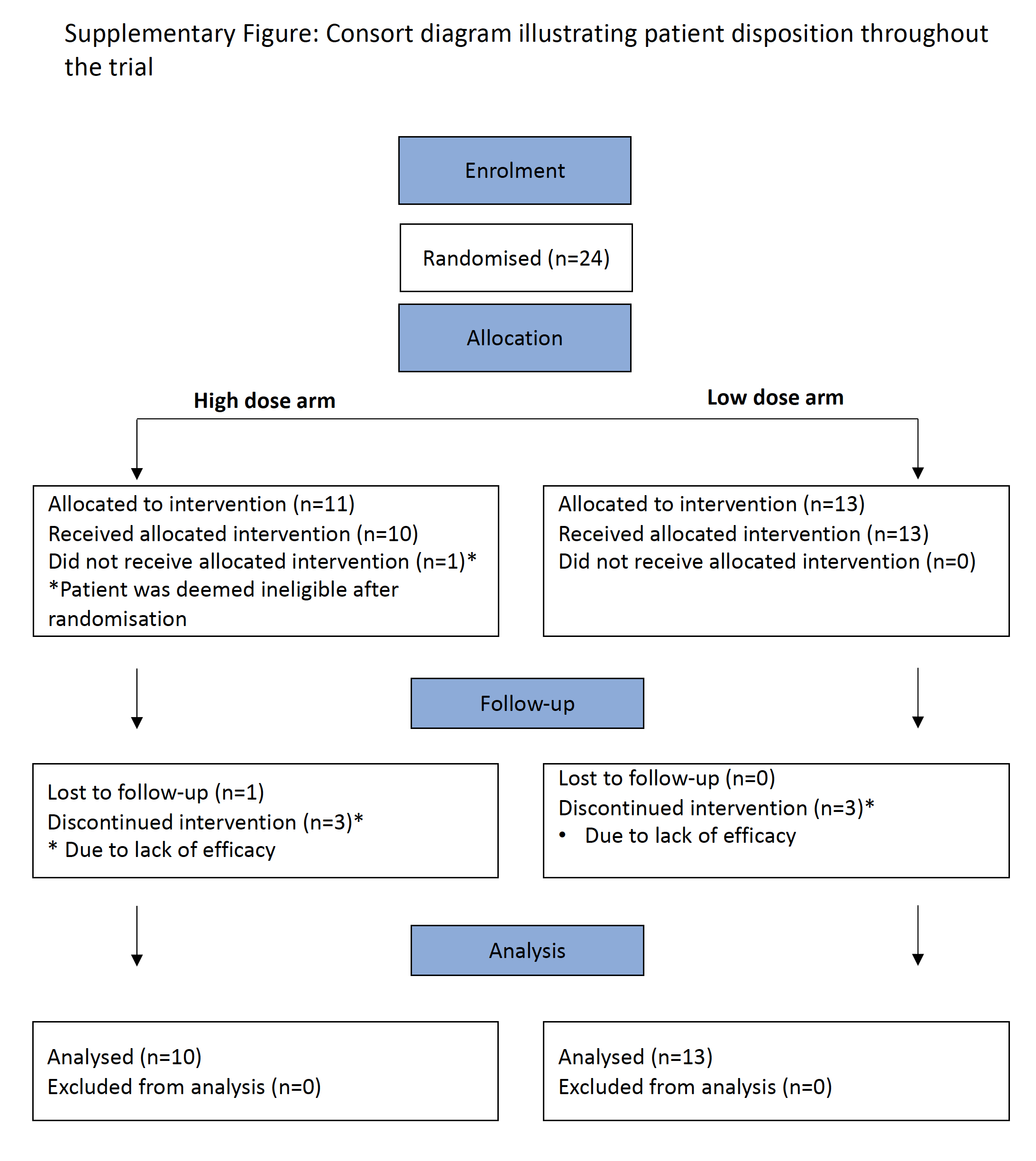

Supplement: Supplementary file 1 — Additional file 1: Supplementary Figure. Consort diagram illustrating patient disposition throughout the trial. [file 13075_2022_2761_MOESM1_ESM.docx]
